# Effectiveness of transcutaneous electrical nerve stimulation in improving cognitive function in older adults with cognitive impairment: a systematic review and meta-analysis

**DOI:** 10.3389/fneur.2025.1556506

**Published:** 2025-04-23

**Authors:** Nga Huen Chan, Shamay S. M. Ng

**Affiliations:** ^1^Department of Rehabilitation Sciences, The Hong Kong Polytechnic University, Hung Hom, Hong Kong SAR, China; ^2^Research Centre for Chinese Medicine Innovation, The Hong Kong Polytechnic University, Hung Hom, Hong Kong SAR, China

**Keywords:** cognitive function, older adults, cognitive impairment, transcutaneous electrical nerve stimulation, meta-analysis

## Abstract

**Background:**

Transcutaneous electrical nerve stimulation (TENS), which involves the application of electrical stimulation to peripheral nerves, is used to improve or maintain cognitive function. Although many studies have examined the effect of TENS on cognition over the past 20 years, a comprehensive review and meta-analysis on this topic is lacking. This study aimed to evaluate the efficacy of TENS in improving cognitive function in older adults with cognitive impairment.

**Methods:**

A systematic search was performed in six electronic databases (CINAHL, Cochrane Library, Embase, Medline, PubMed, and Web of Science) to identify relevant studies published until May 2024. Moreover, the registered clinical trials, forward citation searches, and reference lists of identified publications were reviewed to identify additional relevant studies. Randomised controlled trials investigating the effect of TENS on cognitive function in older adults with cognitive impairment were included.

**Results:**

Seven studies including 247 older adults with cognitive impairment were included. The findings revealed a trend towards positive effects of TENS on face recognition memory [mean difference (MD) = 1.19, 95% confidence interval (CI) = −0.13 to 2.52] and verbal fluency [standardised MD (SMD) = 0.29, 95% CI = −0.01 to 0.59] when compared with placebo stimulation (control condition). TENS demonstrated a significant positive delayed effect on visual memory (SMD = 0.55, 95% CI = 0.11 to 0.98). Subgroup analysis indicated that TENS applied on the concha was more effective than that applied on the spinal column and earlobe in improving verbal memory in the delayed condition.

**Conclusion:**

A positive trend of immediate effect and a significant long-term effect on some cognitive domains were found after applying TENS in in older adults with cognitive impairment. Future studies with robust experimental designs and adequate sample sizes are warranted to investigate the efficacy of TENS in improving cognitive function.

**Systematic review registration:**

https://www.crd.york.ac.uk/PROSPERO/view/CRD42023408611, PROSPERO: CRD42023408611.

## 1 Introduction

Cognitive impairment is defined as substantial impairment in one or more cognitive domains in the fifth edition of the *Diagnostic and Statistical Manual of Mental Disorders* (1). The key domains of cognitive function include perceptual-motor function, language, learning and memory, executive function, complex attention, and social cognition (2). Ageing is a risk factor for cognitive impairment (3). A systematic review published in 2020 revealed that the global prevalence of cognitive impairment amongst older adults was 19% (4). In addition to ageing, chronic diseases, such as stroke (5), diabetes mellitus (6), and chronic kidney disease (7), may cause mild cognitive impairment (MCI) in older adults. MCI is an early stage of symptomatic cognitive decline that does not substantially affect functional ability; however, it can progress to Alzheimer’s disease (AD) (8). Over 60% of individuals with MCI develop AD (9), which adversely affects the quality of life and independence of older adults.

Both pharmacological and nonpharmacological interventions have been proposed to delay cognitive decline and enhance cognitive function in older adults or those with cognitive impairment. However, pharmacological treatments have demonstrated limited effectiveness in improving cognitive impairment and can also lead to some adverse effects (10). Thus, increasing research attention has been focused on nonpharmacological interventions because of their low risk and high generalisability (11).

Transcutaneous electrical nerve stimulation (TENS) or somatosensory stimulation, which involves applying electrical stimulation to peripheral nerves over the thoracic spinal column, has been demonstrated to improve or maintain cognitive function. Previous animal study suggested that peripheral nerve stimulation through TENS activates the hippocampus and increases the release of acetylcholine in the hypothalamus (12). The increased activity in the hippocampus and hypothalamus might prevent cell degeneration in the hippocampus and slows atrophy in the basal forebrain (13), both crucial areas for cognitive processes. TENS applied to the auricular branch of the vagus nerve, known as transcutaneous vagus nerve stimulation (tVNS), has been also demonstrated to improve cognitive function in different populations. Previous functional magnetic resonance imaging (fMRI) studies have demonstrated that tVNS modulates the activities of brain networks, including the brainstem areas, hippocampus, and limbic areas (14, 15), which play crucial roles in memory formation and consolidation, suggesting neural mechanisms through which tVNS enhances cognitive performance.

Two systematic reviews have reported the beneficial effect of TENS on cognitive function (13, 16). Van Dijk et al. (16) reviewed 17 studies investigating the effects of TENS on cognitive and behavioural functioning in various population groups, including those with stroke, AD, and traumatic brain injury. They reported diverse effects of TENS, including the enhancement of somatosensory functioning, visuospatial abilities, and postural control in stroke survivors with neglect and improvement in memory, affective behaviour, and rest–activity rhythm in older adults and individuals with AD (16). However, this review did not synthesise the effect sizes of studies examining improvement in cognitive function. Another meta-analysis by Cameron, Lonergan, and Lee reported a significant positive effect of TENS on face recognition memory (effect size = 2.77, 95% CI = 0.04 to 5.51) after pooling two randomised controlled trials involving individuals with dementia (13). However, the meta-analysis included two studies, and some randomised controlled trials examining the effect of TENS on cognitive function in individuals with other types of cognitive impairment have been published since 2003 (17–20). Thus, an updated systematic review including more recent studies would enhance our understanding of the effect of TENS on cognitive function in individuals with cognitive impairment.

The objective of this systematic review of available randomised controlled trials was to evaluate the efficacy of TENS in improving cognitive function in older adults with cognitive impairment.

## 2 Materials and methods

The review protocol was registered in the PROSPERO database of systematic reviews (registration number: CRD42023408611).

### 2.1 Search strategy

A systematic search was conducted in six electronic databases: CINAHL, Cochrane Library, Embase, Medline, PubMed, and Web of Science. The details of the search strategy used was showed in Supplementary Table 1. The following keywords were used to search articles:

- Randomised controlled trial [MeSH term] OR Controlled clinical trial [MeSH term] OR RCT OR Clinical trial OR Trial OR Intervention OR Therapy
- Ageing [MeSH term] OR Aged [MeSH term] OR Older adults OR Older people OR Elderly
- Cognitive dysfunction [MeSH term] OR Dementia [MeSH term] OR Cognitive impairment OR Cognitive disorder OR Mild cognitive impairment OR Cognitive decline
- Transcutaneous electrical nerve stimulation [MeSH term] OR TENS OR Transcutaneous stimulation OR Electrical stimulation OR Somatosensory stimulation OR Cutaneous electrical stimulation
- Cognition [MeSH term] OR Cognitive function OR Memory [MeSH term] OR Attention [MeSH term] OR Executive function [MeSH term] OR Language [MeSH term] OR Learning [MeSH term]
- 1 AND 2 AND 3 AND 4 AND 5

No restriction regarding the publication date was imposed. Thus, all articles in English language published until May 2024 were considered. To identify additional eligible studies, we reviewed the reference lists of selected studies and registries of clinical trials on ClinicalTrials.gov and examined articles citing the selected studies.

### 2.2 Selection criteria

Studies were included in the review if they (1) were randomised controlled trials, (2) used TENS, (3) included older adults with a mean age of 60 years or above, (4) enrolled participants classified as having cognitive impairment by using the Mini-Mental State Examination (MMSE) or other relevant neuropsychological criteria, and (5) included at least one outcome measure related to cognitive function. Studies were excluded if they (1) did not include a placebo or no-treatment control group, (2) did not report the central tendency and/or variability in the outcome of interest, (3) investigated the effect of electroacupuncture, and (4) applied transcranial electrical stimulation.

### 2.3 Study selection

Two independent reviewers (N.H.C. and P.C.) screened the titles and abstracts of the articles. They evaluated the full texts of potentially relevant articles. If disagreements regarding eligibility occurred, they were resolved through discussion with a third-party reviewer (S.S.M.N.).

### 2.4 Assessment of methodological quality

The methodological quality of each full-text article was evaluated using Version 2 of the Cochrane risk-of-bias tool for randomised trials (RoB 2) (21). The RoB 2 encompasses five domains for assessing the risk of bias: bias arising from randomisation, bias due to deviations from intended interventions, bias due to missing outcome data, bias in the measurement of the outcome, and bias in the selection of reported results (21).

### 2.5 Data extraction

Two reviewers independently extracted data from the included studies. The extracted information included the study design, sample size, population group, age and sex of participants, TENS protocol (frequency, pulse width, intensity, duration of each session, and electrode placement), outcomes related to cognitive function (types of outcome measures and means and standard deviations of the outcomes), and time of measurement. If required data were not published, the study authors were contacted through email, whenever possible, to obtain the data.

The outcome measure of this review was cognitive function. Information on the cognitive domains of working memory, recognition memory (face and picture), visual memory, verbal memory in immediate recall, delayed recall and recognition conditions, verbal fluency, naming ability, inhibitory control, global cognitive function, and executive function was identified and extracted from the included studies.

### 2.6 Statistical analyses

To evaluate the immediate effect of the intervention, the mean change scores of the outcomes were calculated by subtracting the mean score of the baseline assessment from that of the immediate post-intervention assessment. For the delayed effect, the mean change scores of the outcomes were calculated by subtracting the mean score of the baseline assessment from that of the follow-up assessment. The standard deviation (SD) of the mean change scores was imputed using the following formula, with the correlation coefficient (Corr) of 0.8 (22):

$$SD_{\mathrm{change}}=\sqrt{SD_{pre}^{2}+SD_{\mathrm{post}}^{2}-\left(2\times \mathrm{Corr}\times SD_{pre}\times SD_{\mathrm{post}}\right)}$$

Statistical analyses were conducted using Review Manager 5.4 (The Nordic Cochrane Centre, Cochrane Collaboration, Copenhagen, Denmark). Meta-analyses were performed if three or more studies reported the outcome in the same cognitive domain. The effect size in terms of the mean difference (MD) or standardised mean difference (SMD) and its corresponding 95% confidence interval (CI) were computed for all outcomes.

#### 2.6.1 Assessment of heterogeneity

*I^2^* statistics was used to evaluate the heterogeneity of studies. A random-effects model was adopted when the *I^2^* value was greater than 50%, which indicated heterogeneity. A fixed-effects model was used when the *I^2^* value was less than 50% (22).

#### 2.6.2 Publication bias

As only seven studies were included in this review, the sample size was not sufficient to adequately assess funnel plot asymmetry and perform Egger’s regression test, which typically require a minimum of 10 studies to have sufficient power (23). Thus, publication bias was not explored due to the limited number of included studies.

#### 2.6.3 Subgroup analyses

Subgroup analysis was performed to evaluate the effects of different sites of TENS on cognition (i.e., the spinal column, earlobe, and concha).

## 3 Results

### 3.1 Selected studies

**Figure 1:**
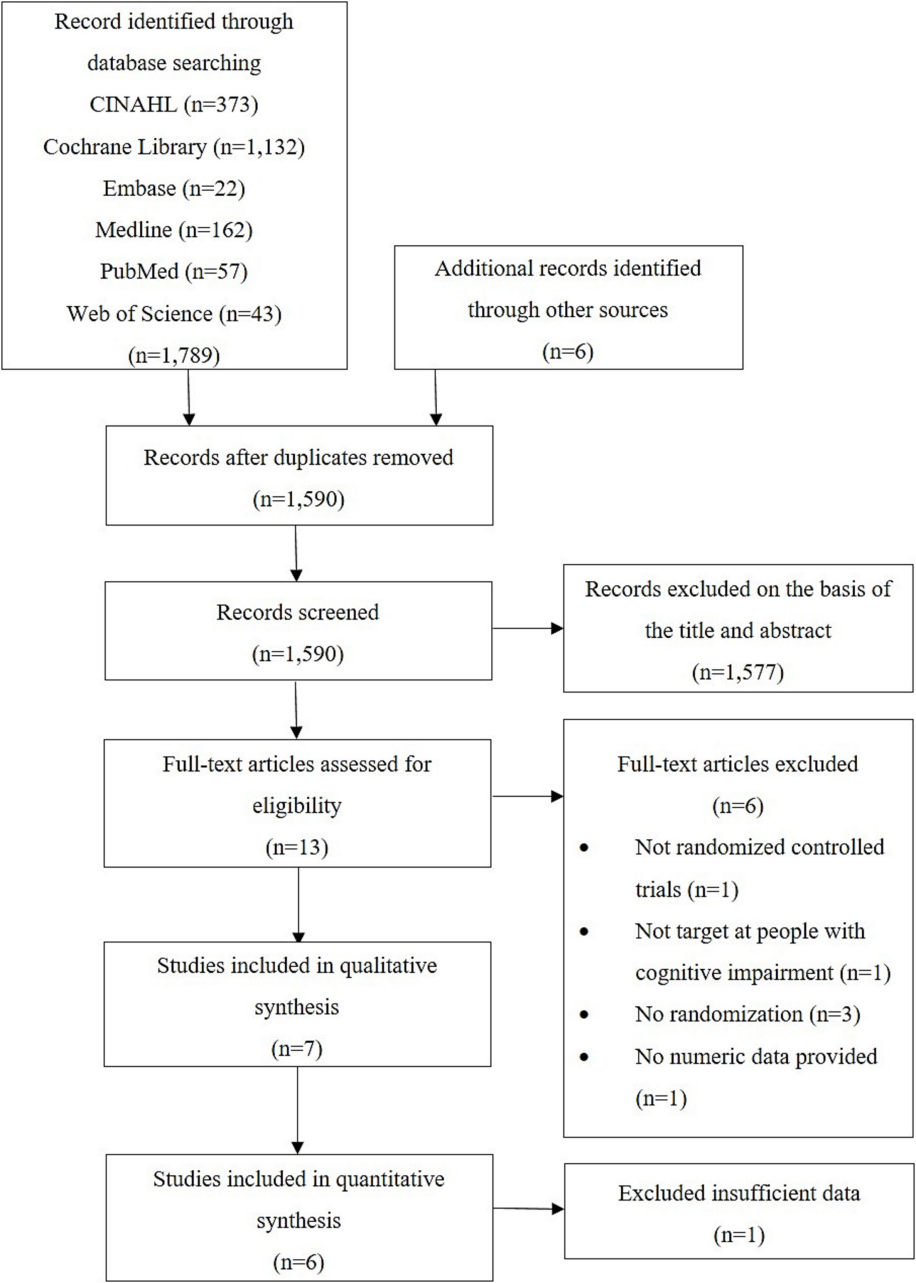
PRISMA flow diagram.

After the removal of duplicates, 1,590 records were identified in the search. Thirteen studies were deemed relevant, and their full texts were assessed for eligibility. Finally, seven studies that met the inclusion criteria were included in our systematic review, and six of them were included in our meta-analysis. The selection process and screening results are summarised in Figure 1.

### 3.2 Study characteristics

The characteristics of the included studies are listed in Supplementary Table 2. Seven randomised controlled trials were eligible for inclusion (17–20, 24–26). The included articles were published between 1998 and 2022. The seven studies included 247 participants with mean ages ranging from 66.9 to 87.9 years. The studies randomly allocated 126 participants into the TENS group and 121 participants into the control group that received placebo or sham stimulation. Two studies recruited patients with MCI (17, 20), four studies included patients with AD, including probable AD (18, 19, 24) and mid-stage AD (26), and one study enrolled older adults who exhibited and/or reported signs of mild forgetfulness (25). Four studies utilised the MMSE, with reported mean scores ranging from 9.4 to 23.4 (17–19, 25), which are lower than the MMSE cut-off score of 24 for detecting cognitive impairment (27). One study used the 12-item MMSE, with a reported mean score of 4.4 (26), which is lower than the cut-off score of 7 for the 12-item MMSE (28). Thus, the participants in this study were classified as having cognitive impairment. Another study used the cognitive screening test (CST) with a reported mean score of 10.4 (24). A score of 12 or below on the CST was used to classify a participant as having cognitive impairment (24). One study indicated that cognitive impairment was diagnosed using the Jak/Bondi’s criteria (29) but did not provide the details of the cognitive screening process. However, this study met our eligibility criteria.

Most of the included studies reported the details of the TENS protocol used in the trials, except the study conducted by Scherder et al. (25). Four studies used a TENS frequency of 160 Hz and a pulse width of 100 μs with an intensity level that evoked painless muscular contraction (17, 19, 24, 26). One study utilised a stimulation frequency of 100 Hz and intensities ranging from 10 to 600 μA (18). Another study applied TENS with a pulse train of 20 Hz for 10 s and 100 Hz for 50 s in each minute and intensities ranging from 0.6 to 1.0 mA (20). The duration of stimulation for each session in all the studies was 30 min. All the studies performed one session of TENS each day, with the exception of one study that conducted two sessions per day (20). The number of sessions per week ranged from 5 to 10. The length of intervention was 6 weeks, in all the studies except one that implemented a 24-week intervention (20). Five studies applied TENS on the spinal column (17, 19, 24–26). In the remaining two studies, electrodes were placed at the earlobes (18) and two auricular acupoints on the concha of the left ear (20).

Six studies conducted 6-week post-intervention assessments to evaluate the long-term effect of TENS on cognitive function (17–19, 24–26). No follow-up assessment was performed in one study (20).

Outcome measures related to different domains of cognitive function and those examined in the included studies are listed in Supplementary Table 3. Five studies assessed working memory using the digit span test (17–19, 24, 26). Six studies examined recognition memory and visual memory using face and picture recognition from the Rivermead Behavioural Memory Test and visual memory span test, respectively (17–19, 24–26). All the studies evaluated verbal memory using the California Verbal Learning Test (17, 25), 8-word test (18, 19, 24, 26), and auditory verbal learning test (20). Furthermore, all the studies assessed verbal fluency using the word fluency task from the Groninger Intelligence Test (17, 18, 24, 26), semantic verbal fluency test (25), animal fluency test (20), and category fluency test (19). One study measured naming ability using the Boston Naming Test, global cognitive function using the Montreal Cognitive Assessment, and executive function using the Shape Trail Test (20). Two studies assessed inhibitory control using the Stroop Color Word Test (19, 25).

### 3.3 Methodological quality

**Figure 2:**
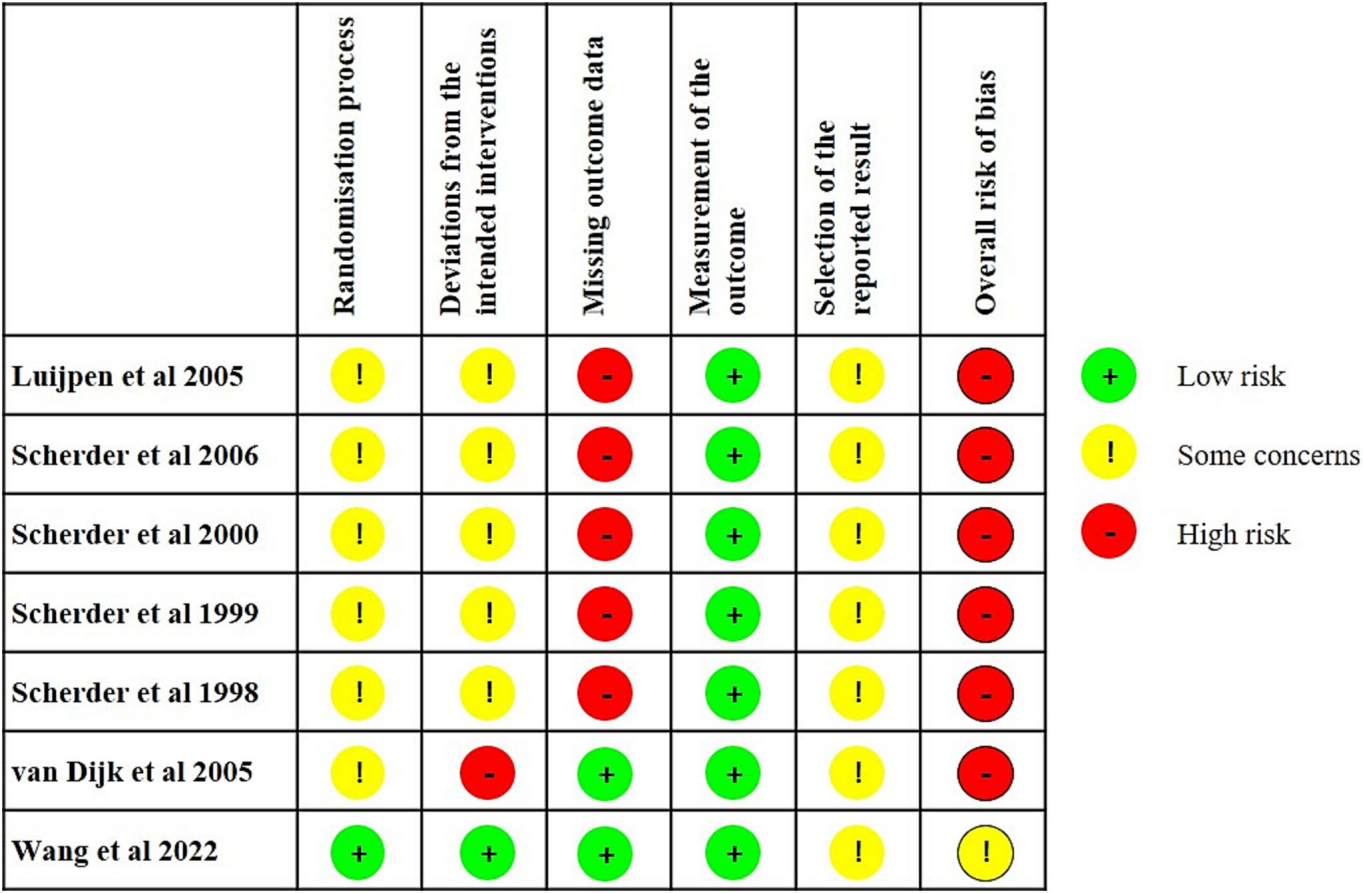
Risk of bias summary: a review of the authors’ judgements about the risk of bias of each item in each included study.

The risk of bias summary is depicted in Figure 2. The signalling questions in RoB 2 and corresponding responses for the evaluation of the risk of bias in the included studies are shown in Supplementary Table 4. Overall, the ratings indicated a high risk of bias in most of the included studies (17–19, 24–26) and some concerns of bias in one study (20). The allocation concealment in six studies was unclear, resulting in some concerns of bias in the randomisation process (17–19, 24–26). One study had a low risk of bias arising from deviations from intended interventions (20). However, five studies had some concerns of bias due to the lack of blinding of research personnel or therapists (17, 18, 24–26). One study had a high risk of bias arising from deviations from intended interventions because of the lack of blinding of therapists delivering the interventions and the use of per-protocol analyses (19). Two studies had a low risk of bias regarding missing outcome data (19, 20) but five studies had a high risk of bias because they lacked information on the extent of missing data (17, 18, 24–26). All studies were considered to have a low risk of bias in the measurement of outcomes. Regarding the selection of reported results, six studies did not include a pre-specified analysis plan (17–19, 24–26) and the planned outcome measurements and analyses differed from those presented in the published report in one study (20). Therefore, all the studies were considered as some concerns of bias due to the risk of reporting bias.

### 3.4 Immediate post-intervention effects of TENS

**Figure 3:**
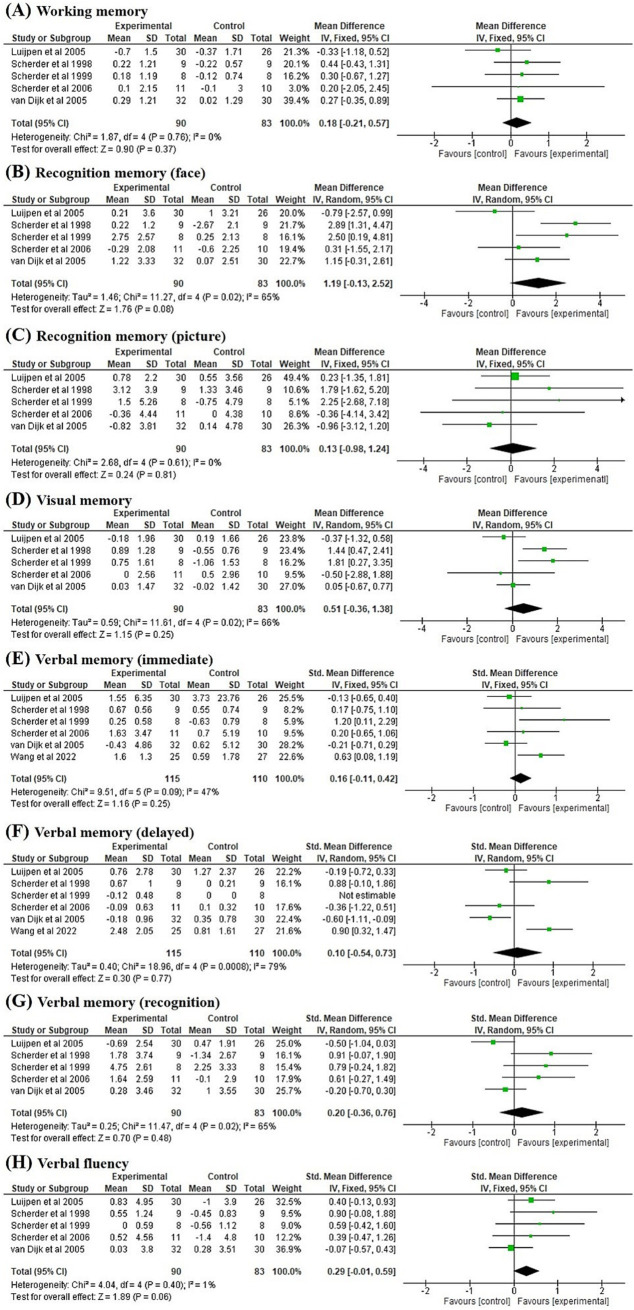
Forest plots of the effects on **(A)** working memory, **(B)** recognition memory (face), **(C)** recognition memory (picture), **(D)** visual memory, **(E)** verbal memory (immediate), **(F)** verbal memory (delayed), **(G)** verbal memory (recognition), and **(H)** verbal fluency compared to control group at immediate post-intervention pooled from six studies.

Figure 3 summarises the immediate post-intervention effect of TENS compared with the control condition. The data provided by Scherder et al. (25) were incomplete. Thus, only six studies were included in the meta-analysis. Wang et al. (20) provided only data related to verbal memory. Thus, six studies (17–20, 24, 26) were pooled in the meta-analysis of verbal memory in immediate and delayed recall conditions (Figures 3E,F). Five studies (24, 26) were pooled in the meta-analysis of working memory (Figure 3A), face recognition memory (Figure 3B), picture recognition memory (Figure 3C), visual memory (Figure 3D), verbal memory in recognition condition (Figure 3G), and verbal fluency (Figure 3H). Our results revealed the effect of TENS on face recognition memory demonstrated a trend towards significance (*p* = 0.08) compared with the control condition (MD = 1.19, 95% CI = −0.13 to 2.52), with significant heterogeneity (*I^2^* = 65%, *p* = 0.02; Figure 3B). The effect of TENS on verbal fluency was marginally significant (*p* = 0.06) compared with the control condition (SMD = 0.29, 95% CI = −0.01 to 0.59), with nonsignificant heterogeneity (*I^2^* = 1%, *p* = 0.40; Figure 3H). No significant effects of TENS were noted on working memory, face recognition memory, picture recognition memory, visual memory, and verbal memory when compared with the control condition.

### 3.5 Subgroup analysis

**Figure 4:**
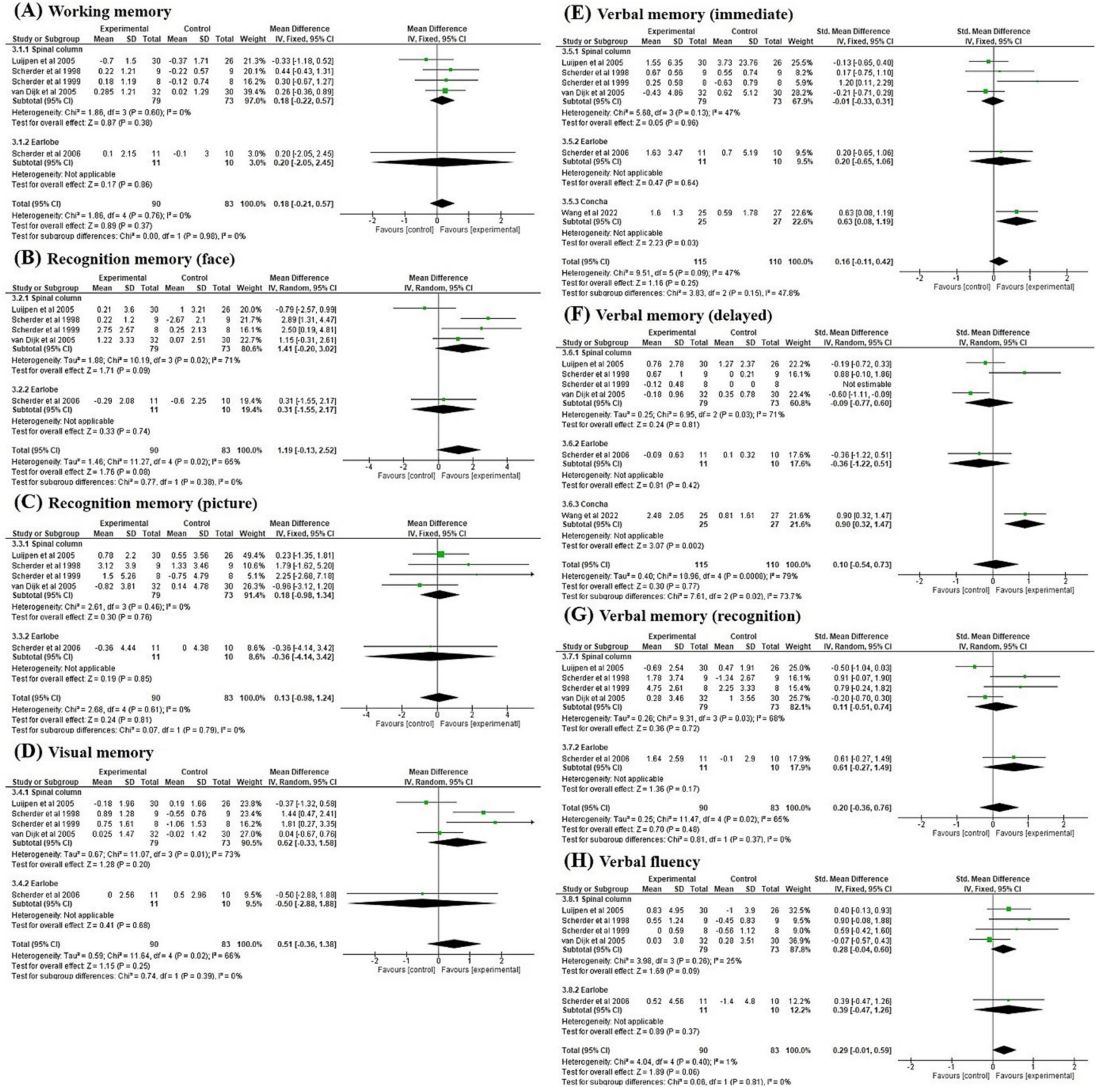
Forest plots for the effects on **(A)** working memory, **(B)** recognition memory (face), **(C)** recognition memory (picture), **(D)** visual memory, **(E)** verbal memory (immediate), **(F)** verbal memory (delayed), **(G)** verbal memory (recognition), and **(H)** verbal fluency at immediate post-intervention by stimulation site of TENS.

The findings of a subgroup analysis performed to investigate the immediate post-intervention effect of TENS applied at different sites are presented in Figure 4. The findings revealed a significant difference in the effect of TENS at different stimulation sites on verbal memory in the delayed condition (*p* = 0.02), with significant heterogeneity (*I^2^* = 79%, *p* < 0.01; Figure 4F). TENS applied on the concha exhibited the largest effect size (SMD = 0.90, 95% CI = 0.32 to 1.47), followed by stimulation on the spinal column (SMD = −0.09, 95% CI = −0.77 to 0.60) and earlobe (SMD = −0.36, 95% CI = −1.22 to 0.51). No significant subgroup differences were noted in working memory, face recognition memory, picture recognition memory, visual memory, verbal memory in immediate recall and recognition conditions, and verbal fluency at different stimulation sites.

### 3.6 Delayed post-intervention effects of TENS

**Figure 5:**
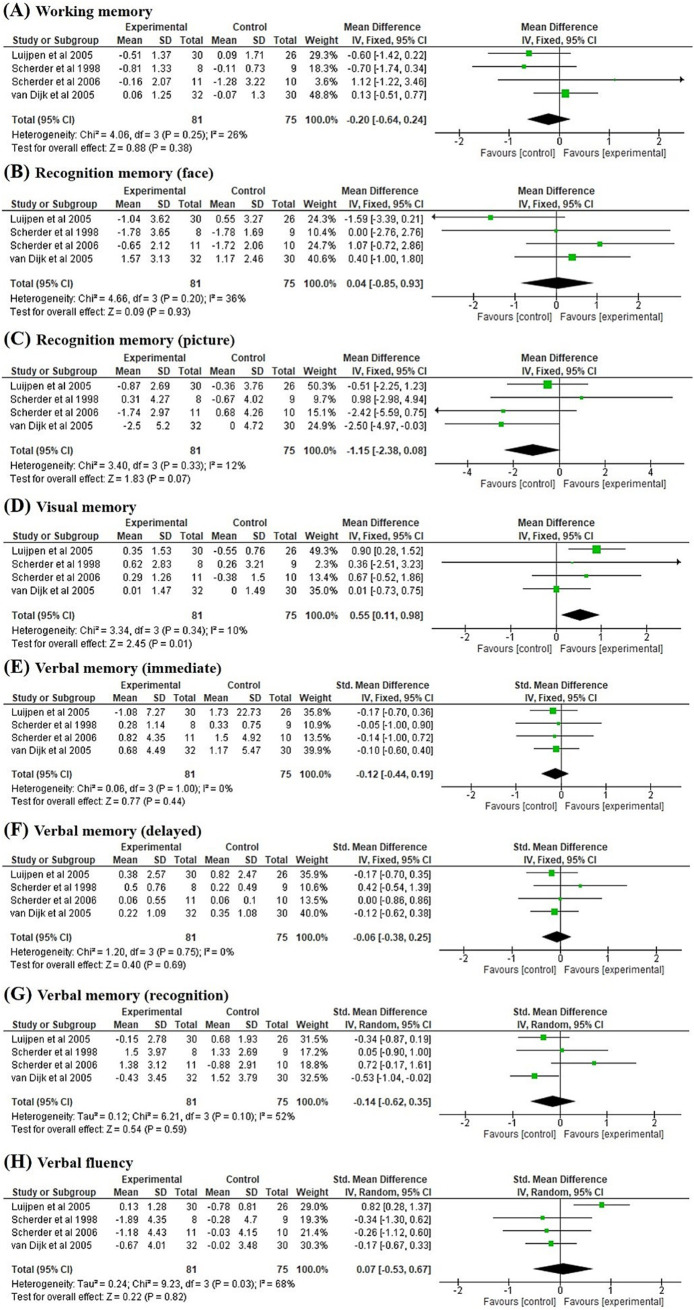
Forest plots of the effects on **(A)** working memory, **(B)** recognition memory (face), **(C)** recognition memory (picture), **(D)** visual memory, **(E)** verbal memory (immediate), **(F)** verbal memory (delayed), **(G)** verbal memory (recognition), and **(H)** verbal fluency compared to control group at 6-week post-intervention pooled from four studies.

Four studies (17–19, 24) investigated the 6-week post-intervention effect of TENS on cognitive function (Figure 5). The effect of TENS on picture recognition memory demonstrated a trend towards significance (*p* = 0.07) in favour of the control condition (MD = −1.15, 95% CI = −2.38 to 0.08), with no significant heterogeneity (*I^2^* = 12%, *p* = 0.33; Figure 5C). Our findings revealed a significant effect of TENS on visual memory (*p* = 0.01) when compared with the control group (SMD = 0.55, 95% CI = 0.11 to 0.98), with no significant heterogeneity (*I^2^* = 10%, *p* = 0.34; Figure 5D). No significant delayed post-treatment effects of TENS were noted on working memory, face recognition memory, picture recognition memory, verbal memory, and verbal fluency.

## 4 Discussion

To the best of our knowledge, this is the first systematic review to investigate the effectiveness of TENS in improving cognitive function in older adults with cognitive impairment. Our results revealed that verbal fluency and face recognition memory had a positive trend of improvement, though insignificant, immediately following the application of TENS. In addition, our findings indicated a significant improvement in visual memory 6 weeks after the cessation of the intervention. Subgroup analyses demonstrated the strongest effect of TENS on verbal memory in the delayed condition when stimulation was applied to the concha. However, these findings need to be considered with caution due to the low certainty of evidence.

The positive trends of treatment effects of TENS on verbal fluency and face recognition memory were observed in patients with cognitive impairment immediately after the intervention. Performance in verbal fluency and face recognition in individuals with cognitive impairment was related to the hippocampus, and some types of dementia cause hippocampal atrophy and dysfunction (30, 31). Afferent signals induced by TENS may regulate the activity of neurotransmitters through ascending neural pathways and stimulate the hippocampus and forebrain through the locus coeruleus and dorsal raphe nucleus (17, 32). This activity aligns with the ‘use it or lose it’ principle, which suggests that such stimulation prevents cell degeneration or facilitates regeneration in the hippocampus (13), thus improving performance in verbal fluency and face recognition. In this study, although TENS appears to improve verbal fluency and face recognition memory in individuals with cognitive impairment, the exact mechanism underlying this effect remains unclear because no neurophysiological or neuroimaging studies have been conducted to investigate its mechanism associated with cognitive improvement. Also, the lack of significant effects of TENS on verbal fluency and face recognition memory may be due to the limited number of included studies coupled with high heterogeneity in stimulation protocols, which warrants the need for further exploratory studies in this context.

A subgroup analysis revealed that TENS applied on the concha was more effective than stimulation on the spinal column and earlobe in terms of improving verbal memory in the delayed recall condition. The delayed recall condition measures the active retrieval of information from verbal memory (19), relying exclusively on the sematic and mnemonic networks of the brain (33). A recent fMRI study revealed that TENS applied on the concha, which is tVNS, significantly increased functional connectivity between critical structures in the sematic network, including the temporal poles, supramarginal gyrus, superior temporal gyrus, and anterior cingulate, compared with sham stimulation (34), by modulating the activity of various neurotransmitters such as norepinephrine, serotonin, dopamine, gamma-aminobutyric acid, and acetylcholine (35). This finding might explain the significant improvement in verbal memory in the delayed recall condition following tVNS. In addition, one of the included studies applied TENS on the earlobe and showed insignificant effect on cognition when compared with the control group (18). The ear lobe, a landmark typically used as sham-stimulation in tVNS studies, is considered to be devoid of auricular innervation (36). A neuroimaging study in 2019 showed that stimulation on the earlobe only produced somatosensory signal response in the postcentral gyrus representation of the face without significant activation in other regions of the brain (37). It is plausible that stimulation on the earlobe may not activate cortical, subcortical, and cerebellar brain regions associated with the afferent vagal pathway. However, the power of the analysis was low due to the small number of trials included. The subgroup analysis was performed with a small number of studies, of which only one study was included in a subgroup to evaluate the effect of TENS on concha and earlobe. The results of the subgroup analysis may be biased by a single included study. Therefore, the findings should be interpreted with caution.

Our results revealed no significant effect of TENS on visual memory immediately after the treatment but its significant effect was observed at 6 week after TENS intervention. All the included studies utilised the visual memory span test to examine the performance of visual working memory, which is related to multiple cognitive processes, such as perception, short-term memory, and attention (38). The improvement in visual memory may be associated with the TENS-induced increase in the activation of the hippocampus and release of acetylcholine in the hypothalamus (12), which play crucial roles in memory, learning, and attention (39). The exact mechanisms underlying the delayed effect of TENS on visual memory remain to be elucidated. Reis et al. suggested that non-invasive electrical stimulation exerted substantial offline effects on skill learning compared with sham stimulation, implying that electrical stimulation affects cognition even after the cessation of stimulation (40). TENS may influence cognitive function by affecting consolidation processes, resulting in a delayed effect on visual memory. Additional studies including follow-up analyses are warranted to confirm the delayed effect of TENS on visual memory.

The effect sizes in this study may have been overestimated due to the high risk of bias in most of the included trials. The low methodological quality of the included studies mainly resulted from unclear allocation concealment and the lack of information on the extent of missing data and prespecified trial protocols. In addition, the lack of blinding of therapists reduced the methodological quality of some studies.

This review has several limitations that need to be addressed. First, the inclusion of only seven studies with small sample sizes might have resulted in low statistical power, limiting the ability to detect the effects of the intervention accurately. The effects of different stimulation protocols on improving cognitive function was also inconclusive due to the limited number of the included studies, which warrants the need for further exploratory studies in this context. Second, the effect sizes may have been overestimated in this meta-analysis due to the high risk of bias in the included studies. Third, high heterogeneity amongst the included studies was observed in the subgroup analysis. This high heterogeneity can be attributed to the inclusion of two types of population groups (AD and MCI) and three stimulation sites for TENS (spinal column, earlobe, and concha) in this study. The treatment effects of TENS may vary amongst different populations and stimulation sites, resulting in higher statistical heterogeneity. Moreover, the use of different stimulation parameters of TENS in the included trials might have influenced treatment effects and resulted in the observed heterogeneity. Future studies should identify the optimal stimulation parameters of TENS to improve cognitive function in individuals with cognitive impairment.

## 5 Conclusion

In this study, a positive trend of immediate effects and significant long-term effects on some cognitive domains were found after applying TENS in individuals with cognitive impairment. In particular, the application of TENS on the concha showed superior results compared with stimulation on the spinal column and earlobe in terms of improving verbal memory in the delayed recall condition. Additional studies with robust experimental designs and large sample sizes are warranted. Although high heterogeneity was observed due to different stimulation parameters in the included studies, these studies can enhance our understanding regarding the effectiveness of TENS in cognitive rehabilitation.

## Data Availability

The original contributions presented in the study are included in the article/Supplementary material, further inquiries can be directed to the corresponding author.
