# Supplementary material for: Effectiveness of transcutaneous electrical nerve stimulation in improving cognitive function in older adults with cognitive impairment: a systematic review and meta-analysis
Source: Front Neurol. 2025 Apr 23;16:1556506. doi: 10.3389/fneur.2025.1556506 (PMC12056508; doi:10.3389/fneur.2025.1556506)
Supplement: Supplementary file 1 [file Supplementary_file_1.docx]

Supplementary Material

# Supplementary Table 1. Search strategy

| **1. Search strategy for CINAHL Complete** | |
| --- | --- |
| TI ( Randomized controlled trial OR Controlled clinical trial OR RCT OR Clinical trial OR Trial OR Intervention OR Therapy ) OR AB ( Randomized controlled trial OR Controlled clinical trial OR RCT OR Clinical trial OR Trial OR Intervention OR Therapy ) | 1,278,288 |
| TI ( Aging OR Aged OR Older adults OR Older people OR Elderly ) OR AB ( Aging OR Aged OR Older adults OR Older people OR Elderly ) | 459,144 |
| TI ( Cognitive dysfunction OR Dementia OR Cognitive impairment OR Cognitive disorder OR Mild cognitive impairment OR Cognitive decline ) OR AB ( Cognitive dysfunction OR Dementia OR Cognitive impairment OR Cognitive disorder OR Mild cognitive impairment OR Cognitive decline ) | 103,996 |
| TI ( Transcutaneous electrical nerve stimulation OR TENS OR Transcutaneous stimulation OR Electrical stimulation OR Somatosensory stimulation OR Cutaneous electrical stimulation ) OR AB ( Transcutaneous electrical nerve stimulation OR TENS OR Transcutaneous stimulation OR Electrical stimulation OR Somatosensory stimulation OR Cutaneous electrical stimulation ) | 383,201 |
| TI ( Cognition OR Cognitive function OR Memory OR Attention OR Executive function OR Language OR Learning ) OR AB ( Cognition OR Cognitive function OR Memory OR Attention OR Executive function OR Language OR Learning ) | 433,484 |
| S1 AND S2 AND S3 AND S4 AND S5 | 373 |
| **2. Search strategy for Cochrane Library** | |
| (Randomized controlled trial OR RCT OR Clinical trial OR Trial OR Controlled clinical trial OR Intervention OR Therapy):ti,ab,kw | 1,619,541 |
| (Aging OR Aged OR Older adults OR Older people OR Elderly):ti,ab,kw | 938,382 |
| (Cognitive dysfunction OR Dementia OR Cognitive impairment OR Cognitive disorder OR Mild cognitive impairment OR Cognitive decline):ti,ab,kw | 72,861 |
| (Transcutaneous electrical nerve stimulation OR TENS OR Transcutaneous stimulation OR Electrical stimulation OR Somatosensory stimulation OR Cutaneous electrical stimulation):ti,ab,kw | 54,227 |
| (Cognition OR Cognitive function OR Memory OR Attention OR Executive function OR Language OR Learning):ti,ab,kw | 201,574 |
| #1 AND #2 AND #3 AND #4 AND #5 | 1,150 |
| Limit #6 to Trials | 1,132 |
| **3. Search strategy for Embase** | |
| 'randomized controlled trial':ti,ab,kw OR rct:ti,ab,kw OR 'clinical trial':ti,ab,kw OR trial:ti,ab,kw OR 'controlled clinical trial':ti,ab,kw OR intervention:ti,ab,kw OR therapy:ti,ab,kw | 5,286,988 |
| aging:ti,ab,kw OR aged:ti,ab,kw OR 'older adults':ti,ab,kw OR 'older people':ti,ab,kw OR elderly:ti,ab,kw | 1,749,978 |
| 'cognitive dysfunction':ti,ab,kw OR dementia:ti,ab,kw OR 'cognitive impairment':ti,ab,kw OR 'cognitive disorder':ti,ab,kw OR 'mild cognitive impairment':ti,ab,kw OR 'cognitive decline':ti,ab,kw | 351,768 |
| 'transcutaneous electrical nerve stimulation':ti,ab,kw OR tens:ti,ab,kw OR 'transcutaneous stimulation':ti,ab,kw OR 'electrical stimulation':ti,ab,kw OR 'somatosensory stimulation':ti,ab,kw OR 'cutaneous electrical stimulation':ti,ab,kw | 89,061 |
| cognition:ti,ab,kw OR 'cognitive function':ti,ab,kw OR memory:ti,ab,kw OR attention:ti,ab,kw OR 'executive function':ti,ab,kw OR language:ti,ab,kw OR learning:ti,ab,kw | 2,008,557 |
| #1 AND #2 AND #3 AND #4 AND #5 | 22 |
| **4. Search strategy for Medline** | |
| TI ( Randomized controlled trial OR Controlled clinical trial OR RCT OR Clinical trial OR Trial OR Intervention OR Therapy ) OR AB ( Randomized controlled trial OR Controlled clinical trial OR RCT OR Clinical trial OR Trial OR Intervention OR Therapy ) | 4,376,312 |
| TI ( Aging OR Aged OR Older adults OR Older people OR Elderly ) OR AB ( Aging OR Aged OR Older adults OR Older people OR Elderly ) | 1,276,051 |
| TI ( Cognitive dysfunction OR Dementia OR Cognitive impairment OR Cognitive disorder OR Mild cognitive impairment OR Cognitive decline ) OR AB ( Cognitive dysfunction OR Dementia OR Cognitive impairment OR Cognitive disorder OR Mild cognitive impairment OR Cognitive decline ) | 271,291 |
| TI ( Transcutaneous electrical nerve stimulation OR TENS OR Transcutaneous stimulation OR Electrical stimulation OR Somatosensory stimulation OR Cutaneous electrical stimulation ) OR AB ( Transcutaneous electrical nerve stimulation OR TENS OR Transcutaneous stimulation OR Electrical stimulation OR Somatosensory stimulation OR Cutaneous electrical stimulation ) | 458,007 |
| TI ( Cognition OR Cognitive function OR Memory OR Attention OR Executive function OR Language OR Learning ) OR AB ( Cognition OR Cognitive function OR Memory OR Attention OR Executive function OR Language OR Learning ) | 1,570,699 |
| S1 AND S2 AND S3 AND S4 AND S5 | 162 |
| **5. Search strategy for PubMed** | |
| (Randomized controlled trial[MeSH Terms]) OR (Controlled clinical trial[MeSH Terms]) OR (RCT OR Clinical trial OR Trial OR Intervention OR Therapy) | 1,778,637 |
| (Aging[MeSH Terms]) OR (Aged[MeSH Terms]) OR (Older adults[Title/Abstract] OR Older people[Title/Abstract] OR Elderly[Title/Abstract]) | 380,728 |
| (Cognitive dysfunction[MeSH Terms]) OR (Dementia[MeSH Terms]) OR (Cognitive impairment[Title/Abstract] OR Cognitive disorder[Title/Abstract] OR Mild cognitive impairment[Title/Abstract] OR Cognitive decline[Title/Abstract]) | 30,358 |
| (Transcutaneous electrical nerve stimulation[MeSH Terms]) OR (TENS[Title/Abstract] OR Transcutaneous stimulation[Title/Abstract] OR Electrical stimulation[Title/Abstract] OR Somatosensory stimulation[Title/Abstract] OR Cutaneous electrical stimulation[Title/Abstract]) | 8,113 |
| (Cognition[MeSH Terms]) OR (Cognitive function[Title/Abstract]) OR (Memory[MeSH Terms]) OR (Attention[MeSH Terms]) OR (Executive function[MeSH Terms]) OR (Language[MeSH Terms]) OR (Learning[MeSH Terms]) | 88,666 |
| #1 AND #2 AND #3 AND #4 AND #5 | 57 |
| **6. Search strategy for Web of Science** | |
| (TI=(Randomized controlled trial OR Controlled clinical trial OR RCT OR Clinical trial OR Trial OR Intervention OR Therapy)) OR (AB=(Randomized controlled trial OR Controlled clinical trial OR RCT OR Clinical trial OR Trial OR Intervention OR Therapy) | 4,672,286 |
| (TI=(Aging OR Aged OR Older adults OR Older people OR Elderly )) OR AB=(Aging OR Aged OR Older adults OR Older people OR Elderly ) | 4,349,961 |
| (TI=(Cognitive dysfunction OR Dementia OR Cognitive impairment OR Cognitive disorder OR Mild cognitive impairment OR Cognitive decline )) OR AB=(Cognitive dysfunction OR Dementia OR Cognitive impairment OR Cognitive disorder OR Mild cognitive impairment OR Cognitive decline ) | 372,967 |
| (TI=(Transcutaneous electrical nerve stimulation OR TENS OR Transcutaneous stimulation OR Electrical stimulation OR Somatosensory stimulation OR Cutaneous electrical stimulation )) OR AB=(Transcutaneous electrical nerve stimulation OR TENS OR Transcutaneous stimulation OR Electrical stimulation OR Somatosensory stimulation OR Cutaneous electrical stimulation ) | 147,422 |
| (TI=(Cognition OR Cognitive function OR Memory OR Attention OR Executive function OR Language OR Learning )) OR AB=(Cognition OR Cognitive function OR Memory OR Attention OR Executive function OR Language OR Learning ) | 4,429,383 |
| #1 AND #2 AND #3 AND #4 AND #5 | 43 |

# Supplementary Table 2. Characteristics of the included studies.

| **Study** | **Sample size (TENS/control)** | **Participant** | **Mean age of participant** | **TENS parameters** | **Assessment of cognitive function** | **Time of measurement** | **Results** |
| --- | --- | --- | --- | --- | --- | --- | --- |
| Luijpen et al., 2005 | 30/26 | People with MCI (mean MMSE score = 22.4 – 23.4) | Experimental group: 87.3 ± 5.6; Control group: 87.2 ± 4.8 | *Frequency:* 160 Hz  *Pulse width:* 100 μs  *Intensity:* Evoke painless muscular contraction  *Stimulation duration:* 30 mins/session; 7 days/week for 6 weeks  *Electrode placement:* Both sides of spinal column between T1 and T5 | 1. Digit span test; 2. Visual memory span test; 3. The verbal learning and memory test; 4. Face recognition from the RBMT; 5. Picture recognition from the RBMT; 6. Word fluency from the GIT | Pre-treatment baseline, second baseline, post-intervention, and 6-week post-intervention | TENS did not improve cognitive function. |
| Scherder et al., 2006 | 11/10 | People with probable AD (mean MMSE score = 18.0 – 20.0) | Experimental group: 83.7; Control group: 84.5 | *Frequency:* 100 Hz  *Pulse width:* Not mentioned  *Intensity:* 10 to 600 μA  *Stimulation duration:* 30 mins/session, 5 days/week for 6 weeks  *Electrode placement:* Earlobes | 1. Digit span test; 2. Visual memory span test; 3. The 8 words test; 4. Face recognition of the RBMT; 5. Picture recognition of the RBMT; 6. Word fluency from the GIT. | Baseline, post-intervention, and 6-week post-intervention | TENS did not improve cognitive function. |
| Scherder et al., 2000 | 10/10 | Older adults who showed and/or reported signs of mild forgetfulness (mean MMSE score = 9.4 – 9.7) | Experimental group: 85.9; Control group: 87.9 | *Frequency:* Not mentioned  *Pulse width:* Not mentioned  *Intensity:* Not mentioned  *Stimulation duration:* 30 mins/session, 5 days/week for 6 weeks  *Electrode placement:* Both sides of spinal column between T1 and T5 | 1. Visual memory span test; 2. Recall and recognition of the CVLT; 3. Face recognition of the RBMT; 4. Picture recognition of the RBMT; 5. Semantic verbal fluency; 6. Stroop color word test | Baseline, post-intervention, and 6-week post-intervention | TENS improved visual memory span test score, CVLT total score, performance in face recognition of the RBMT and semantic verbal fluency relative to sham-TENS. |
| Scherder et al., 1999 | 9/9 | People with midstage AD (mean 12-item MMSE score = 4.4) | 81.7 | *Frequency:* 160 Hz  *Pulse width:* 100 μs  *Intensity:* Evoke painless visible muscular contraction  *Stimulation duration:* 30 mins/session; 5 days/week for 6 weeks  *Electrode placement:* Both sides of spinal column between T1 and T5 | 1. Digit span test; 2. Visual memory span test; 3. The 8 words test; 4. Face recognition of the RBMT; 5. Picture recognition of the RBMT; 6. Word fluency from the GIT. | Baseline, post-intervention, and 6-week post-intervention | TENS improved visual memory span test score relative to sham-TENS, but the effect did not persisted at 6 weeks post-intervention. |
| Scherder et al., 1998 | 9/9 | People with probable AD (mean cognitive screening test = 10.4) | 83.4 | *Frequency:* 160 Hz  *Pulse width:* 100 μs  *Intensity:* Evoke painless visible muscular contraction  *Stimulation duration:* 30 mins/session, 5 days/week for 6 weeks  *Electrode placement:* Both sides of spinal column between T1 and T5 | 1. Digit span test; 2. Visual memory span test; 3. The 8 words test; 4. Face recognition of the RBMT; 5. Picture recognition of the RBMT; 6. Word fluency from the GIT. | Baseline, post-intervention, and 6-week post-intervention | TENS improved visual memory span test score, performance in face recognition of the RBMT and word fluency from the GIT relative to sham-TENS. The effects in visual memory span test and word fluency from the GIT persisted at 6 weeks post-intervention. |
| van Dijk et al., 2005 | 32/30 | People with probable AD (mean MMSE score = 15.2) | Experimental group: 71.0 ± 7.8; Control group: 72.5 ± 8.2 | *Frequency:* 160 Hz  *Pulse width:* 100 μs  *Intensity:* Evoke painless visible muscular contraction  *Stimulation duration:* 30 mins/session; 7 days/week for 6 weeks  *Electrode placement:* Both sides of spinal column between T1 | 1. Digit span test; 2. Visual memory span test; 3. The 8 words test; 4. Face recognition of the RBMT; 5. Picture recognition of the RBMT; 6. Stroop color word test; 7. Category fluency test | Baseline, post-intervention, and 6-week post-intervention | TENS did not improve cognitive function. |
| Wang et al., 2022 | 25/27 | People with MCI | Experimental group: 66.9 ± 3.7; Control group: 67.0 ± 4.4 | *Frequency:* 20 Hz for 10 seconds and 100 Hz for 50 s in each minute  *Pulse width:* Not mentioned  *Intensity:* 0.6 to 1.0 mA  *Stimulation duration:* 30 mins/session; 2 sessions/day; 5 days/week for 24 weeks  *Electrode placement:* Concha of the left ear | 1. MoCA; 2. Auditory verbal learning test; 3. Shape trails test; 4. AFT; 5. Boston naming test | Baseline and post-intervention | TENS improved MoCA score and AFT immediate and delayed recall scores relative to sham-TENS. |
| AD: Alzheimer's disease; AFT: Animal fluency test; CVLT: California Verbal Learning Test; GIT: Groninger Intelligence Test; MCI: mild cognitive impairment; MMSE: Mini-Mental Status Examination; MoCA: Montreal cognitive assessment; RBMT: Rivermead Behavioural Memory Test; TENS: transcutaneous electrical nerve stimulation. | | | | | | | |

# Supplementary Table 3. Outcome measures related to different cognitive function in the included studies.

|  | **Global cognitive function** | **Working memory** | **Face recognition memory** | **Picture recognition memory** | **Visual memory** | **Verbal memory** | **Verbal fluency** | **Naming ability** | **Inhibitory control** | **Executive function** |
| --- | --- | --- | --- | --- | --- | --- | --- | --- | --- | --- |
| **Luijpen et al., 2005** |  | Digit span test | Face recognition from the RBMT | Picture recognition from the RBMT | Visual memory span test | California Verbal Learning Test | Word fluency from the GIT |  |  |  |
| **Scherder et al., 2006** |  | Digit span test | Face recognition from the RBMT | Picture recognition from the RBMT | Visual memory span test | The 8 words test | Word fluency from the GIT |  |  |  |
| **Scherder et al., 2000** |  |  | Face recognition from the RBMT | Picture recognition from the RBMT | Visual memory span test | California Verbal Learning Test | Semantic verbal fluency test |  | Stroop color word test |  |
| **Scherder et al., 1999** |  | Digit span test | Face recognition from the RBMT | Picture recognition from the RBMT | Visual memory span test | The 8 words test | Word fluency from the GIT |  |  |  |
| **Scherder et al., 1998** |  | Digit span test | Face recognition from the RBMT | Picture recognition from the RBMT | Visual memory span test | The 8 words test | Word fluency from the GIT |  |  |  |
| **van Dijk et al., 2005** |  | Digit span test | Face recognition from the RBMT | Picture recognition from the RBMT | Visual memory span test | The 8 words test | Category fluency test |  | Stroop color word test |  |
| **Wang et al., 2022** | Montreal cognitive assessment |  |  |  |  | Auditory verbal learning test | Animal fluency test | Boston naming test |  | Shape trails test |
| ; GIT: Groninger Intelligence Test; RBMT: Rivermead Behavioural Memory Test. | | | | | | | | | | |

**Supplementary Table 4. Signalling questions in RoB 2 and corresponding responses for the evaluation of risk of bias in the included studies.**

|  | **Luijpen et al 2005** | **Scherder et al 2006** | **Scherder et al 2000** | **Scherder et al 1999** | **Scherder et al 1998** | **van Dijk et al 2005** | **Wang et al 2022** |
| --- | --- | --- | --- | --- | --- | --- | --- |
| *Domain 1: Bias arising from randomisation* | *Some concerns* | *Some concerns* | *Some concerns* | *Some concerns* | *Some concerns* | *Some concerns* | *Low risk* |
| 1.1 Was the allocation sequence random? | NI | Y | NI | NI | NI | Y | Y |
| 1.2 Was the allocation sequence concealed until participants were enrolled and assigned to interventions? | NI | NI | NI | NI | NI | NI | Y |
| 1.3 Did baseline differences between intervention groups suggest a problem with the randomization process? | N | N | NI | N | N | N | N |
| *Domain 2: Bias due to deviations from intended interventions* | *Some concerns* | *Some concerns* | *Some concerns* | *Some concerns* | *Some concerns* | *High risk* | *Low risk* |
| 2.1. Were participants aware of their assigned intervention during the trial? | PN | NI | NI | N | PN | N | N |
| 2.2. Were carers and people delivering the interventions aware of participants' assigned intervention during the trial? | PY | PY | PY | PY | PY | Y | N |
| 2.3. If Y/PY/NI to 2.1 or 2.2: Were there deviations from the intended intervention that arose because of the trial context? | NI | NI | NI | NI | NI | / | / |
| 2.4 If Y/PY to 2.3: Were these deviations likely to have affected the outcome? | / | / | / | / | / | NI | N |
| 2.5. If Y/PY/NI to 2.4: Were these deviations from intended intervention balanced between groups? | / | / | / | / | / | / | / |
| 2.6 Was an appropriate analysis used to estimate the effect of assignment to intervention? | Y | Y | Y | Y | Y | N | / |
| 2.7 If N/PN/NI to 2.6: Was there potential for a substantial impact (on the result) of the failure to analyse participants in the group to which they were randomized? | / | / | / | / | / | / | / |
| *Domain 3: Bias due to missing outcome data* | *High risk* | *High risk* | *High risk* | *High risk* | *High risk* | *Low risk* | *Low risk* |
| 3.1 Were data for this outcome available for all, or nearly all, participants randomized? | NI | NI | NI | NI | N | N | N |
| 3.2 If N/PN/NI to 3.1: Is there evidence that the result was not biased by missing outcome data? | N | N | N | N | N | N | N |
| 3.3 If N/PN to 3.2: Could missingness in the outcome depend on its true value? | NI | NI | NI | NI | NI | N | N |
| 3.4 If Y/PY/NI to 3.3: Is it likely that missingness in the outcome depended on its true value? | NI | NI | NI | NI | NI | N | N |
| *Domain 4: Bias in the measurement* *of the outcome* | *Low risk* | *Low risk* | *Low risk* | *Low risk* | *Low risk* | *Low risk* | *Low risk* |
| 4.1 Was the method of measuring the outcome inappropriate? | N | N | N | N | N | N | N |
| 4.2 Could measurement or ascertainment of the outcome have differed between intervention groups? | N | N | N | N | N | N | N |
| 4.3 If N/PN/NI to 4.1 and 4.2: Were outcome assessors aware of the intervention received by study participants? | N | N | NI | N | N | N | NI |
| 4.4 If Y/PY/NI to 4.3: Could assessment of the outcome have been influenced by knowledge of intervention received? | / | / | N | / | / | / | N |
| 4.5 If Y/PY/NI to 4.4: Is it likely that assessment of the outcome was influenced by knowledge of intervention received? | / | / | NI | / | / | / | NI |
| *Domain 5: Bias in the selection of reported results* | *Some concerns* | *Some concerns* | *Some concerns* | *Some concerns* | *Some concerns* | *Some concerns* | *Some concerns* |
| 5.1 Were the data that produced this result analysed in accordance with a pre-specified analysis plan that was finalized before unblinded outcome data were available for analysis? | NI | NI | NI | NI | NI | NI | N |
| 5.2 Is the numerical result being assessed likely to have been selected, on the basis of the results, from multiple eligible outcome measurements (e.g. scales, definitions, time points) within the outcome domain? | NI | NI | NI | NI | NI | NI | NI |
| 5.3 Is the numerical result being assessed likely to have been selected, on the basis of the results, from multiple eligible analyses of the data? | NI | NI | NI | NI | NI | NI | NI |
| PN: Probably no, PY: Probably yes, N: No, NI: No information, Y: Yes. | | | | | | | |
